# Traumatic chylothorax management post-coronary artery bypass grafting – A systematic review

**DOI:** 10.1177/02184923251321541

**Published:** 2025-02-23

**Authors:** Gavin John Carmichael, Duron Prinsloo, Connor Bentley, Rodan Prinsloo, Joshua G Kovoor, Mathew O Jacob, Aashray Gupta

**Affiliations:** 1 2281 University of Melbourne, Melbourne, Victoria, Australia; 2 637124 Grampians Health, Ballarat, Victoria, Australia; 3 3376 Western Hospital, Footscray, Victoria, Australia; 4 St Vincent's Hospital, Fitzroy, Victoria, Australia; 5 2113 Cairns and Hinterland Hospital and Health Service, Cairns, Queensland, Australia; 6 Department of Surgery, University of Adelaide, Adelaide, South Australia, Australia

**Keywords:** myocardial revascularization, coronary artery bypass, chylothorax, complication, general, aortic

## Abstract

**Introduction:**

Coronary artery bypass graft (CABG) surgery is performed globally around 400,000 times annually. Despite its benefits, CABG can lead to complications, including chylothorax, a rare condition where chyle accumulates in the pleural cavity due to thoracic duct trauma. Currently, there are no international guidelines for traumatic chylothorax management post-CABG. This is the first systematic review to provide a comprehensive overview of the current state of management for chylothorax post-CABG.

**Methods:**

This systematic review was conducted by searching EMBASE, Cochrane, Ovid and PubMed databases on 16 June 2024. The inclusion criteria focused on studies addressing post-CABG chylothorax management and reporting clinical outcomes. Data was extracted from 11 studies focusing on graft type, complications and management strategies.

**Results:**

This review included 11 case report studies with 14 cases of post-CABG chylothorax. Conservative management was attempted in all cases, with varying components such as total parenteral nutrition, nil by mouth, octreotide and low-fat diets. High-output chylothorax (>1000 mL/day) often necessitated surgical intervention after an average of 12.5 days of conservative management. Surgical approaches included thoracic duct ligation, embolisation and pleurodesis. Surgical ligation was effective in three cases, while thoracic duct embolisation was successful in one case.

**Conclusions:**

Chylothorax post-CABG is managed initially with conservative strategies, but high-output cases often necessitate surgical intervention. This review highlights the need for standardised guidelines, regarding the timing of surgical escalation and the use of octreotide and somatostatin. Further research should focus on higher-powered studies to validate these findings and establish clinical guidelines for managing chylothorax post-CABG.

## Introduction

Chylothorax is a rare complication of coronary artery bypass (CABG) surgery and presents a particularly challenging problem. Chylothorax forms when chyle, a milky bodily fluid consisting of lymph, phospholipids, long-chain triglycerides and cholesterol, accumulates in the pleural cavity due to trauma of the thoracic duct.^1^

The aetiology of chylothorax can be classified into four categories: congenital, traumatic, neoplastic and miscellaneous.^2^ In the context of CABG, the most relevant category is traumatic chylothorax. Traumatic chylothorax is notably associated with damage incurred to the thoracic duct during harvesting of the left internal mammary artery (LIMA).^2^

The incidence of chylothorax post-CABG surgery is relatively low, reported to be approximately 0.038%.^3^ However, when it does occur, it can lead to significant morbidity due to nutritional deficiencies, immune suppression and prolonged hospital stays.^4^ Risk factors for developing chylothorax include extensive mediastinal dissection, re-operative surgery and anatomical variations in the thoracic duct.^5^

The current gold standard for coronary revascularisation is grafting of the LIMA. The LIMA is preferred not only over venous grafts but also over other arterial grafts due to its superior patency rates, with a reported >90% ten-year patency.^6^ However, given its anatomical proximity to the thoracic duct, harvesting the LIMA increases the risk of thoracic duct injury and subsequent chylothorax.^1^ As an adjunct, or when the LIMA is unsuitable, the right internal mammary artery (RIMA) is also considered in appropriate patients.^7^

Chylothorax can be further categorised into low and high-output types based on the volume of chyle drainage. Low-output chylothorax is typically managed conservatively with dietary modifications, such as a low-fat diet or medium-chain triglycerides, and occasionally, total parenteral nutrition (TPN)^8^ with the aim of reducing chyle production. High-output chylothorax, characterised by drainage exceeding 1000 mL/day, often necessitates more aggressive interventions due to the higher failure rate of medical management.^8^

Currently, there are no specific international guidelines for the management of traumatic chylothorax following CABG surgery, likely due to its low incidence of occurrence. The management strategies are generally extrapolated from broader guidelines on the treatment of chylothorax from other causes, emphasising the need for a standardised approach tailored to post-CABG patients.

This systematic review aims to evaluate the existing management strategies for traumatic chylothorax post-CABG surgery, reflect on current standards of practice and identify gaps in the literature. By synthesising available evidence, this review seeks to provide a comprehensive overview of the management options.

## Methods

### Search strategy

On the 27th of July 2024, a comprehensive search was performed on MEDLINE (EMBASE), MEDLINE (Ovid), PubMed and Cochrane electronic databases. A combination of keyword terms and medical subject headings (MeSH) were used in the searches. The publications included were dated from 2013 to June 2024 using the search terms ‘chylothorax’ OR ‘coronary artery bypass graft surgery’ OR ‘CABG’ OR ‘post-operative chylothorax’ as keywords or MeSH. The included articles were screened, and their reference lists were checked manually.

### Eligibility criteria

Studies that looked at the management of Chylothorax complication Post-Coronary Artery Bypass Grafting.

Inclusion criteria consisted of:

(i) Post-operative chylothorax (ii) post-CABG (iii) Written in English (iv) Clinical Outcomes (v) Management strategy and (vi) Articles since January 2013

Exclusion criteria consisted of:

(i) Nil clinical outcomes (ii) Not written in English (iii) Review articles (iv) Conference Abstracts (v) Posters and (vi) Articles before January 2013

### Study selection

Eligibility of articles was assessed by two independent reviewers. PRISMA guidelines were followed.^9^ Disagreements were resolved by consensus. A review protocol was not prepared, and this systematic review was not submitted to PROSPERO as there is a limited amount of literature, majority being low level.

Article titles and abstracts were initially screened. The remaining articles had a full-text review, and articles were included or excluded based upon the eligibility criteria. Any discrepancies were resolved by consensus. Covidence was utilised.

### Risk of bias assessment

Limited by the incidence of chylothorax, all articles included in this review were case reports. The articles were assessed for risk of bias using the Joanna Briggs Institute Critical Appraisal tools for use in JBI Systematic Reviews – Checklist for Case Reports^10^ (see Table 1). No studies were excluded during this process.

**Table 1.** JBI critical appraisal checklist for case reports.

Authors	Were patient's demographic characteristics clearly described?	Was the patient's history clearly described and presented as a timeline?	Was the current clinical condition of the patient on presentation clearly described?	Were diagnostic tests of assessment methods and results clearly described?	Was the intervention(s) or treatment procedure(s) clearly described?	Was the post-intervention clinical condition clearly described?	Were adverse events (harms) or unanticipated events identified and described?	Does the case report provide takeaway lessons?
El-Farra et al.^14^ Case Report (Level 4)	No	No	No	No	Yes	Yes	Unclear	Yes
Khan et al.^17^ Case Report (Level 4)	Yes	Yes	Yes	Yes	Yes	Yes	Yes	Yes
Waliany et al.^22^ Case Report (Level 4)	Yes	No	Yes	Yes	Yes	Yes	Yes	Yes
Waikar et al.^21^ Case Report (Level 4)	No	Yes	Yes	Yes	Yes	Yes	Yes	Yes
Mukherjee et al.^18^ Case Report (Level 4)	No	No	No	Yes	Yes	Yes	Yes	Yes
Deguchi et al.^13^ Case Report (Level 4)	Yes	Yes	Yes	Yes	Yes	Yes	Yes	Yes
Altun et al.^12^ Case Report (Level 4)	No	No	Yes	Yes	Yes	Yes	Yes	Yes
Kanna et al.^16^ Case Report (Level 4)	Yes	Yes	Yes	Yes	Yes	Yes	Yes	Yes
Vazhev et al.^20^ Case Report (Level 4)	No	No	Yes	Yes	Yes	Yes	Yes	Yes
Sabzi et al.^19^ Case Report (Level 4)	Yes	Yes	Yes	Yes	Yes	Yes	Yes	Yes
Hoskote et al.^15^ Case Report (Level 4)	Yes	No	Yes	Yes	Yes	Yes	Yes	No

### Data extraction

Data was summarised from the included studies using a data collection spreadsheet in Excel. The spreadsheet contained: Intervention, vessel bypassed, vessel harvested (LIMA/RIMA), survival rate, complications, days from surgery to diagnosis, chylous chest drain volume, triglyceride levels, medical management of chylothorax (TPN, nil by mouth [NBM], octreotide/somatostatin, oral diet and length of medical management) and surgical management.

## Results

### Search results

**Figure 1.**
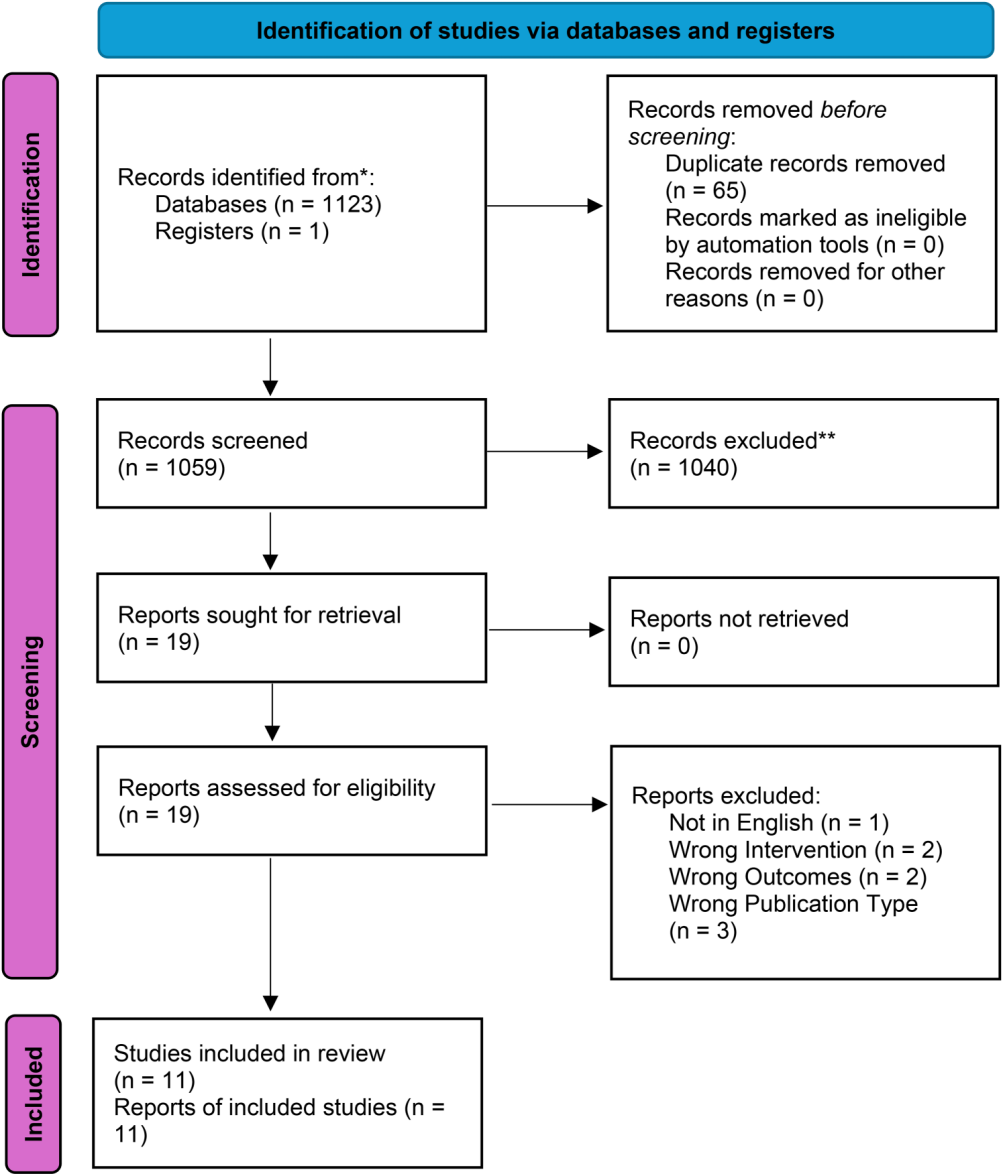
PRISMA flow diagram.

The search of MEDLINE (EMBASE), Cochrane, MEDLINE (Ovid) and PubMed revealed 1124 articles. Using Covidence,^11^ 65 duplicates were identified and removed, 1059 articles were available for screening. Of these, 1040 did not meet the inclusion criteria after title and abstract screening. The remaining 19 articles were examined in-depth with a full-text review, of which 11 met the criteria for inclusion in the systematic review.^12–22^ Figure 1 demonstrates the PRISMA flow diagram^9^ of the study selection process.

### Study characteristics

The included 11 studies, published between 2013 and 2021, examined 14 patients with chylothorax following CABG. The characteristics and interventions are detailed in Table 2. Ages ranged from 41 to 82 years, with majority of patients being male (9 out of 15). Left internal mammary artery grafting was the predominant technique (11 of 14 cases), while RIMA was used in one case;^13^ two papers did not report on their conduit of choice.^15,16^ Two studies had operative variations which included concomitant valve replacements.^14,20^ Follow-up durations ranged from 30 days to 1 year, with limited data on long-term recurrence.

**Table 2.** Patient characteristics.

Author, date, journal, study type (level of evidence)	Participants	Sex + Age	Intervention	LIMA/ RIMA	Follow-up period
El-Farra et al. (2021), Ann Thorac Surg^14^ Case Report (Level 4)	2	60M (Patient 1)	4-vessel CABG	LIMA	NR
		61F (Patient 2)	4-vessel CABG + MVR	LIMA	NR
Khan et al. (2020), J Saudi Heart Assoc.^17^ Case Report (Level 4)	1	41M	4-vessel CABG	LIMA	30 days
Waliany et al. (2018), Journal of Cardiothoracic Surgery. ^22^ Case Report (Level 4)	1	62M	4-vessel CABG	LIMA	2- and 8-weeks
Waikar et al. (2018), Ann Card Anaesth. ^21^ Case Report (Level 4)	1	52M	3-vessel CABG	LIMA	30 days
Mukherjee et al. (2016), Indian J Chest Dis Allied Sci. ^18^ Case Report (Level 4)	2	56M (Patient 1)	4-vessel CABG	LIMA	3 months and 1 year
		50M (Patient 2)	3-vessel CABG	LIMA	3 months and 1 year
Deguchi et al. (2015), Gen Thorac Cardiovasc Surg. ^13^ Case Report (Level 4)	1	78F	3-vessel CABG	RIMA	3 and 6 months
Altun et al. (2015), Tex Heart Inst J.^12^ Case Report (Level 4)	2	60M (Patient 1)	2-vessel CABG	LIMA	1 year
		46M (Patient 2)	3-vessel CABG	LIMA	1 year
Kanna et al. (2021), J Card Surg. ^16^ Case Report (Level 4)	1	63F	3-vessel CABG	NR	6 months
Vazhev et al. (2020), Acta Medica Bulgarica. ^20^ Case Report (Level 4)	1	56M	1-vessel CABG + AVR + MVR	LIMA	NR
Sabzi et al. (2016), J Cardiovasc Thorac Res. ^19^ Case Report (Level 4)	1	68F	3-vessel CABG	LIMA	6 months
Hoskote et al. (2013), Blood Coagul Fibrinolysis.^15^ Case Report (Level 4)	1	82F	5-vessel CABG	NR	NR

AVR: aortic valve replacement; CABG: coronary artery bypass grafting; F: female; LIMA: left internal mammary artery; M: male; MVR: mitral valve replacement; NR: not reported; RIMA: right internal mammary artery.

### Low-output chylothorax

Regarding sole conservative management, three out of six low-output chylothorax cases^12,19^ (<1000 mL/day) were managed this way, as summarised in Table 3. Drainage volumes ranged from 450 to 770 mL/day, with triglyceride levels consistently above 300 mg/dL. Survival rate for these cases was 100%. The time from post-operative day to diagnosis ranged from 1 to 5 days. Breast necrosis was reported in one case.^19^

**Table 3.** Low-output chylothorax: diagnosis, other complications, survival rate and management principles.

Author, date, journal, study type (level of evidence)	Chylous chest drain volume (mL)	Triglyceride level (mg/dL)	POD to diagnosis (days)	Other complications	Survival rate (%)	Management (conservative/ surgical/both)
El-Farra et al. (2021), Ann Thorac Surg ^14^ Case Report (Level 4)	500–600 (Patient 1)	NR	4	Nil	100	Both
Mukherjee et al. (2016), Indian J Chest Dis Allied Sci. ^18^ Case Report (Level 4)	450 (Patient 1)	852	3	Nil	100	Both
Altun et al. (2015), Tex Heart Inst J.^12^ Case Report (Level 4)	NR (Patient 1)	398	2	Nil	100	Conservative
	NR (Patient 2)	276	3	Nil	100	Conservative
Vazhev et al. (2020), Acta Medica Bulgarica. ^20^ Case Report (Level 4)	770	NR	5	Nil	100	Both
Sabzi et al. (2016), J Cardiovasc Thorac Res. ^19^ Case Report (Level 4)	NR	320	1	Breast necrosis	100	Conservative

POD: post-operative day; NR: not reported.

### High-output chylothorax

High-output chylothorax cases (>1000 mL/day) demonstrated greater variability in management and outcomes, as detailed in Table 4. Drain volumes ranged from 1500 to 2400 mL/day^17,21^ with triglyceride levels ranging from 65 mg/dL^13^ to over 1600 mg/dL.^22^ Five of the eight cases^13,14,18,21,22^ transitioned to surgical intervention following failed conservative management, with an average delay of 12.5 days (range: 4–30 days). One case^16^ used surgical management only. Two cases^15,17^ utilised conservative treatment only. Regarding survival rate, one out of eight cases died,^15^ with pulmonary embolism as the cause of death.

**Table 4.** High-output chylothorax: diagnosis, other complications, survival rate and management principles.

Author, date, journal, study type (level of evidence)	Chylous chest drain volume (mL)	Triglyceride level (mg/dL)	POD to diagnosis (days)	Other complications	Survival rate (%)	Management (conservative/ surgical/both)
El-Farra et al. (2021), Ann Thorac Surg ^14^ Case Report (Level 4)	2000 (Patient 2)	NR	2	Nil	100	Both
Khan et al. (2020), J Saudi Heart Assoc.^17^ Case Report (Level 4)	2400	NR	3	Nil	100	Conservative
Waliany et al. (2018), Journal of Cardiothoracic Surgery. ^22^ Case Report (Level 4)	2300	1604	13	Yellow Nail Syndrome	100	Both
Waikar et al. (2018), Ann Card Anaesth. ^21^ Case Report (Level 4)	2400	448	6	Nil	100	Both
Mukherjee et al. (2016), Indian J Chest Dis Allied Sci. ^18^ Case Report (Level 4)	1500 (Patient 2)	1169	14	Nil	100	Both
Deguchi et al. (2015), Gen Thorac Cardiovasc Surg. ^13^ Case Report (Level 4)	2140	65	3	Nil	100	Both
Kanna et al. (2021), J Card Surg. ^16^ Case Report (Level 4)	1800	1043	14	Nil	100	Surgical
Hoskote et al. (2013), Blood Coagul Fibrinolysis.^15^ Case Report (Level 4)	1500	555	14 days (Post D/C)	Pulmonary embolism	0	Conservative

POD: post-operative day; NR: not reported; D/C: discharge.

### Conservative management

Conservative management strategies varied significantly, as summarised in Table 5. Common components included TPN, NBM and dietary modifications, with octreotide or somatostatin used in seven cases.^12–14,16,17,21,22^ The dosage of octreotide ranged from 100 µg to 300 µg, with the treatment duration tailored to the patient's specific conservative management plan, ranging from 5 to 30 days. Somatostatin treatment was initiated at a dose of 3.5 µg, escalated to 5 µg after 48 h and increased to 7 µg after an additional 48 h, with total treatment duration of 6 days.

**Table 5.** Conservative management of chylothorax post-CABG.

Author, date, journal, study type (level of evidence)	TPN	NBM	Octreotide/ Somatostatin	Oral Diet Step-down	Length of Conservative Mx (days)
El-Farra et al. (2021), Ann Thorac Surg ^14^ Case Report (Level 4)	Yes (Patient 1)	Yes	IV, dose NR	No	10
	Yes (Patient 2)	Yes	No	No	14
Khan et al. (2020), J Saudi Heart Assoc.^17^ Case Report (Level 4)	Yes	Yes	No	Low-fat diet	7
Waliany et al. (2018), Journal of Cardiothoracic Surgery. ^22^ Case Report (Level 4)	Yes	Yes	Dose NR	Low-fat diet w/ MCFA	18
Waikar et al. (2018), Ann Card Anaesth. ^21^ Case Report (Level 4)	Yes with low-fat MCFA 1500 kcal/day restriction	Yes	Octreotide 100 units TDS SC	No	4
Mukherjee et al. (2016), Indian J Chest Dis Allied Sci. ^18^ Case Report (Level 4)	No (Patient 1)	No	Octreotide 100 µg TDS SC 200 µg TDS SC (day 6)	Low-fat MCFA	30
	No (Patient 2)	No	Octreotide 100 µg TDS SC	Low-fat MCFA	30
Deguchi et al. (2015), Gen Thorac Cardiovasc Surg. ^13^ Case Report (Level 4)	Yes	Yes	Octreotide 300 µg daily IV	No	5
Altun et al. (2015), Tex Heart Inst J.^12^ Case Report (Level 4)	Low-fat + MCFA 1800 kcal/day restriction	Yes	Somatostatin IV 3.5 µg/kg/h for first 48 h 5 µg/kg/h for following 48 h 7 µg/kg/h for following 48 h	Oral low-fat diet (day 7)	11
	Low-fat + MCFA 1800 kcal/day restriction	Yes	As above	Oral low-fat diet (day 7)	12
Kanna et al. (2021), J Card Surg. ^16^ Case Report (Level 4)	High protein + MCFA	Yes	Octreotide 100 µg TDS SC (day 7) Octreotide continuous IV infusion (day 8)	No	6
Vazhev et al. (2020), Acta Medica Bulgarica. ^20^ Case Report (Level 4)	No	No	No	No-fat diet (day 1)	10
Sabzi et al. (2016), J Cardiovasc Thorac Res. ^19^ Case Report (Level 4)	Yes	Yes	No	MCFA	8

IV: intravenous; MCFA: medium-chain fatty acid; Mx: management; NBM: nil by mouth; NR: not reported; SC: subcutaneous; TDS: three times a day; TPN: total parenteral nutrition.

### Surgical and interventional management

Surgical and interventional outcomes are summarised in Table 6. Pleurodesis was performed in six cases, with mixed results. Talc pleurodesis was unsuccessful in one case,^18^ while doxycycline pleurodesis resolved chylothorax following talc failure.^22^ Surgical ligation, employed in three cases,^13,14,20^ was guided by lesion visualization techniques such as lymphangiography^20^ and nasogastric milk administration.^13^ Thoracic duct embolization (TDE), performed in one case,^14^ was successful in sealing the leak using platinum coils and *n*-Butyl-2-cyanoacrylate.

**Table 6.** Surgical and interventional management.

Author, date, journal, study type (level of evidence)	Re-admission (Yes/No?)	Lesion visualised?		POD of surgery (days)	Surgical and interventional Mx
		Yes/No	Investigation		
El-Farra et al. (2021), Ann Thorac Surg^14^ Case Report (Level 4)	No (Patient 1)	No	Lymphangiogram	14	Embolisation with platinum coils + nBCA
	No (Patient 2)	Yes	Redo median sternotomy	17	Surgical ligation
Waliany et al. (2018), Journal of Cardiothoracic Surgery.^22^ Case Report (Level 4)	Yes	Yes	Lymphangiogram	33	Thoracoscopic pleurodesis
				38	Doxycycline pleurodesis
Waikar et al. (2018), Ann Card Anaesth.^21^ Case Report (Level 4)	No	No	Nil	11	Povidone-iodine pleurodesis
Mukherjee et al. (2016), Indian J Chest Dis Allied Sci.^18^ Case Report (Level 4)	No (Patient 1)	No	Nil	33	Talc pleurodesis
	Yes (Patient 2)	No	Nil	43	Talc pleurodesis
Deguchi et al. (2015), Gen Thorac Cardiovasc Surg.^13^ Case Report (Level 4)	No	Yes	Median sternotomy with NG milk administration	10	Surgical ligation technique and clipping
Kanna et al. (2021), J Card Surg.^16^ Case Report (Level 4)	Yes	Yes	Diagnostic thoracoscopy	>60	Talc pleurodesis
Vazhev et al. (2020), Acta Medica Bulgarica.^20^ Case Report (Level 4)	No	Yes	Left lateral thoracotomy	13	Surgical ligation and pleurodesis

N/A: not available.

## Discussion

### Conservative management

#### Efficacy and variability of conservative management

The relatively short average duration of conservative management reported in the reviewed studies (see Table 5) suggests that, in specific patient demographics, conservative measures can effectively resolve chylothorax without necessitating surgical intervention. However, conflicting evidence exists regarding the efficacy of conservative management, particularly in cases of high-output, traumatic chylothoraces.^4,23,24^ Prior literature has recommended surgical intervention for high-output chylothorax (defined as >1100 mL/day) due to the increased likelihood of conservative management failure.^25^ Nonetheless, individual cases, such as the one described by Khan et al.^17^ (Table 5), demonstrate the potential for successful conservative treatment even in high-output scenarios.

In this case, a patient with a chest drain volume of 2400 mL/day achieved resolution after 7 days of conservative management, which included TPN, NBM and an oral step-down to a low-fat diet on day 4, without complications.

The absence of a standardised guideline for conservative management is evident in the diverse strategies employed across the literature. Different combinations of TPN, NBM, octreotide and dietary modifications have been used, as reported by Altun et al.^12^ and Hoskote et al.^12,15^ (see Table 5). Similarly, Sabzi et al.^19^ described a nine-day conservative approach that included a low-fat diet and an anti-hypertriglyceridemia drug.

The variability in treatment approaches highlights the need for further studies to focus on the combination of conservative treatments to optimise patient outcomes and reduce hospitalisation time. Establishing a standardised guideline for conservative management would improve consistency across different institutions.

### Escalation of intervention

Prior literature^25^ on non-CABG procedures causing chylothorax typically recommends escalation to surgical management when chest tube output exceeds 1000 mL/day. However, the literature in this review suggests a general preference for initially attempting conservative management, with surgery considered if there is no adequate response within two weeks. Eight out of 14^13–18,21,22^ patients demonstrated a high-output chylothorax (>1000 mL on the first day), with two cases using conservative management only, five of these cases had their chylothorax-managed surgery after failed conservative treatment. Only one case^16^ treated the high-output chylothorax with surgical management as first-line therapy. Kanna et al.^16^ utilised first-line surgical intervention due to this patient having a presenting 2 months later due to breathlessness and ultimately a chylothorax. Since a significant time had passed since the initial CABG surgery, video-assisted thoracic surgery was performed due to worries about adhesions surrounding the thoracic duct.^26^

These studies represent the general lack of consensus and consultation within the literature regarding surgical management in high-output chylothoraxes. These results demonstrate and highlight that there is a lack of international guidelines on when to escalate to surgical management immediately or transition from conservative treatment for chylothorax post-CABG. On average, patients underwent approximately 12.5 days of conservative management before proceeding to surgery, with this period ranging from as short as 4 days to as long as 30 days (see Table 5). Prolonged conservative management, even when surgical intervention might be appropriate, can lead to adverse outcomes for patients. Prior literature^27^ indicates that longer hospital admissions can increase patient morbidity, mortality and complications. Therefore, further research is needed to understand the risk ratio of surgical intervention versus the risk of longer hospital admissions to determine the optimal timing for escalation in patient care.

### Octreotide and somatostatin

Octreotide is preferred in treatment protocols over somatostatin due to its longer half-life and the advantage of not requiring continuous infusion.^28^ However, there is no established gold standard for the dosage, frequency and route of administration of octreotide and somatostatin in the management of chylothorax post-CABG. Majority of cases utilised the same dosage and route of administration (ROA) of Octreotide, 100 µg TDS subcutaneously, however, majority of these patients eventually required dose escalation or change to a continuous intravenous infusion. The reasons for these adjustments were not explained in the studies however it could be hypothesised that escalation was needed due to the chylothorax failing to respond.^29^ All patients treated with octreotide ultimately required surgical intervention, whereas those who received somatostatin achieved resolution of their chylothorax through conservative management alone. However, the limited data in this review, coupled with the absence of studies comparing octreotide versus somatostatin in chylothorax, prevents definitive conclusions from being made regarding their comparative efficacy. The high variation in dosage, agent and ROA underscores the inconsistency in conservative management approaches. Rigorous guidelines and high-quality research should aim to determine which specific patient groups benefit most from octreotide versus somatostatin, the appropriate dosage, indications for dose escalation and the link between the use of somatostatin analogues and the need for surgical management.

### Surgical and interventional management

#### Pleurodesis

Pleurodesis was typically performed later in the management process. The rationale for using pleurodesis in delayed presentations of chylothorax is hypothesised to involve the development of adhesions^26^ from CABG-induced trauma, incomplete closure of the lymphatic leak, formation of new leaks post-surgery or failure of other surgical options.^30^ The cases demonstrated that two out of the three patients who re-presented with chylothoraces received conservative treatment first. This contradicts current literature recommendations^26^ for non-CABG chylothorax, which suggest that extended periods post-surgery is an indication of pleurodesis.

A critical issue in the use of pleurodesis is whether lesion visualization should precede the intervention. Some studies advocate for imaging techniques such as lymphangiograms, thoracoscopy or thoracotomy to confirm the site of chyle leakage, while others recommend proceeding based solely on clinical presentation. This inconsistency reflects a lack of consensus and underscores the need for standardized protocols to determine the necessity of lesion visualization. Such guidelines could enhance decision-making by tailoring interventions to patient-specific factors and improving overall outcomes. Further research is warranted to evaluate whether pre-intervention visualization correlates with higher success rates of pleurodesis and reduced recurrence or complications.

Talc pleurodesis is regarded as the standard approach for managing chylothorax following failed embolization, surgical ligation or delated presentation.^30^ However, it does not guarantee resolution in all cases. For example, one study highlighted^22^ the successful use of doxycycline pleurodesis after talc pleurodesis had failed, raising questions about the comparative efficacy of these two approaches. Prior literature^31^ supports this observation reporting a 78% success rate for doxycycline pleurodesis following unsuccessful talc pleurodesis. Studies in other populations, such as children with congenital malformations causing chylothorax,^32^ have demonstrated that doxycycline pleurodesis is a safe and effective first-line therapy. These findings suggest that further research is required to determine the comparative efficacy of talc versus doxycycline pleurodesis post-CABG for the management of chylothorax.

#### Surgical ligation

Surgical ligation is regarded as an effective treatment for chylothorax, particularly in non-CABG settings.^33^ Prior studies have demonstrated its efficacy, with success rates as high as 85% for thoracic duct ligation in cases of iatrogenic postoperative chylothorax.^25^ A key aspect of successful ligation is the ability to localise the site of leakage, typically achieved through lymphangiography.^34^ Interestingly, the reviewed studies involving these techniques did not consistently describe how the lesion was visualised during the procedure. While some studies mentioned methods such as milk administration via nasogastric tube, this was not reported in all cases, leaving uncertainties about the preferred approaches for visualisation.

Furthermore, the use of adjunctive measures, such as mechanical pleurodesis performed immediately after ligation in one study,^20^ raises questions about strategies to prevent recurrence. This practice may address the high reoperation rates associated with surgical ligation, though its rationale and effectiveness are not well documented. There is a need for further research into the procedural aspects of surgical ligation, including techniques for lesion visualisation and the role of adjunctive interventions.

#### Embolisation

First introduced by Cope and Kaiser in 2002, percutaneous thoracic duct embolisation^35^ has emerged as a minimally invasive treatment option for chylothorax, with numerous refinements in the technique since its inception.^24,36^ A 2018 meta-analysis documented a 79.4% for TDE in traumatic chylothorax, highlighting its potential as an effective intervention.^37^ While the reviewed studies included only two cases of TDE use in post-CABG chylothorax, the findings suggest that it can manage chyle leaks^38^ even in the absence of direct leakage visualisation. El-Farra et al.^14^ reported no visualisation of a leakage but instead retrograde reflux of contrast from the upper thoracic duct on a lymphangiogram.

Institutional preferences also play a significant role in the choice of intervention. For instance, Waliany et al.^22^ emphasised their preference for embolisation over ligation due to institution-specific superior outcomes and less invasive nature. This preference expresses the growing appeal of TDE as a first-line or adjunctive option in managing chylothorax, particularly for patients who may not tolerate more invasive procedures. Further research is needed to establish standardized guidelines for when and how TDE should be incorporated into the management of post-CABG chylothorax.

### Left internal mammary artery versus RIMA

The choice of graft in CABG, particularly between the LIMA and RIMA, may influence the risk of developing postoperative chylothorax. This review suggests a higher incidence of chylothorax following the use of LIMA compared to RIMA. However, this apparent discrepancy must be interpreted with caution due to a lack of data on the total frequency of LIMA and RIMA grafts. The observed higher incidence with LIMA could simply reflect its more frequent use rather than an intrinsic higher risk.

The anatomical differences between LIMA and RIMA grafts may contribute to variations in chylothorax risk. Damage to thymic lymphatic channels during LIMA harvesting, particularly when electrocautery is used, has been hypothesised to increase the likelihood of chyle leakage.^39^ This is supported by previous suggestions that electrocautery may exacerbate lymphatic leakage due to its impact on lymphostasis.^19^ Conversely, the lower incidence of RIMA grafting, often due to clinical factors such as insufficient blood flow, might account for the reduced reports of chylothorax associated with this graft. Ultimately, the relationship between graft choice and chylothorax risk remains unclear without data comparing the overall frequency and outcomes of LIMA versus RIMA grafts.

### Multifactorial, patient-centred approach

The varied outcomes and approaches underscore the importance of a multifactorial, patient-centred strategy in managing chylothorax. Decisions regarding the timing and type of surgical intervention are highly individualised, considering the patient's response to conservative management, the visualisation of the lesion and overall clinical status. Overall, the data suggests that while conservative management is often the first line of treatment, a significant proportion of patients will eventually require surgical intervention. The choice and timing of surgery should be tailored to the individual patient, with a preference for less invasive options like embolisation when feasible. However, the potential need for pleurodesis later in the management process indicates that even successful initial surgeries may not be definitive, necessitating continued monitoring and potential further intervention. This comprehensive, patient-centred approach is essential for optimising outcomes in chylothorax management.

## Limitations/bias/future directions

A limitation of this systematic review is the potential for publication bias due to the restriction of articles to the English language. Additionally, the inclusion of articles ranging from 2013 to 2024 was selected to reflect contemporary management practices, but this may have excluded relevant studies published outside this time frame. The exclusion of conference abstracts, presentations and posters may have also omitted pertinent data.

### Case reports

There are major constraints in the literature presented. Chief among these is the style of published articles. Much of the discourse surrounding chylothorax after CABG is based upon low-level literature – meaning that confounding factors and potential biases are not controlled for. This presents major limitations particularly in terms of the ability to comment on the validity and efficacy of management options performed in the treatment of chylothorax post-CABG. Whilst the current management dogma presented in these case reports broadly resulted in the resolution of the chylothorax, due to the small sample sizes of the reports, it remains unclear whether one management approach was more effective than another, or if one management approach resulted in faster resolution of the chylothorax in comparison to another.

#### Medical history

Another major limitation is the lack of patient medical history provided in the studies presented. Unreported pathology could potentially impact patient susceptibility to chylothorax, as well as the choice of management, and therefore act as a potential confounder. Similarly, two of the patients in our review underwent surgical valvular replacement and CABG simultaneously;^14,20^ factors such as these may also have confounded results.

#### Publication bias

Of the 14 reported cases of chylothorax in our review, the in-hospital mortality rate was 7% (see Tables 3 and 4). Interestingly, this is inconsistent with previous literature that has shown the in-hospital mortality rate to be approximately 17%.^40^ Whilst our smaller sample size could account for this discrepancy, we cannot discount the tendency for favourable outcomes to be reported on and the confounding effect this reporting/publication bias may have on the literature.

These limitations necessitate the need for higher power studies with larger sample sizes to minimise potential confounding factors, validate findings and guide the establishment of clinical guidelines.

### Future directions

To facilitate the establishment of standardised clinical guidelines for the management of chylothorax post-CABG, future research should focus on several key areas.

Firstly, in terms of conservative management, future research should focus on factors that influence the choice between somatostatin and octreotide, drug dosing and indications for dose escalation.

Regarding surgical management, a risk ratio comparing the surgical risk and length of hospital admission should be undertaken to determine the optimal timing of escalation to surgery. Additionally, future research should compare the safety and efficacy of surgical and interventional options – with a focus on longer term recurrence of chylothorax and complications.

Out of the 14 patients in this review, six required pleurodesis. Therefore, future studies should better characterise which patient populations would benefit from pleurodesis and the optimal timing of this procedure – particularly in relation to the other management approaches. Additionally, the efficacy of talc versus doxycycline pleurodesis within the context of chylothorax post-CABG should also be explored.

Thus, we recommend that higher power studies are undertaken to overcome the previously mentioned limitations and help establish standardised clinical guidelines. However, the low incidence rate of chylothorax post-CABG may present a significant barrier to these efforts, and therefore, any future research will likely need to be multicentre in nature to attempt to overcome this issue.

## Conclusion

This systematic review highlights the complexity and variability in managing chylothorax following CABG surgery. While conservative management remains the initial approach for most cases, the review indicates that high-output chylothorax often necessitates surgical intervention, with a preference for techniques such as thoracic duct ligation and embolisation. The variability in conservative management practices, particularly regarding the use of octreotide and somatostatin, highlights a critical need for standardised guidelines.

Overall, the findings emphasise the necessity for more robust, multi-institutional studies to better understand the optimal timing and choice of interventions. Establishing clearer guidelines will not only improve patient care but also reduce the variability in treatment approaches observed across different institutions. However, given the rarity of post-CABG traumatic chylothorax, the feasibility of developing formal clinical guidelines is limited. The rarity of this condition constrains such high-level research, making the construction of comprehensive guidelines impractical. Future research should aim to generate shared clinical insights and consensus-based recommendations through multi-institutional collaboration and case series analysis. While these will not constitute formal guidelines or protocols, they can help establish practical frameworks for managing this rare but significant complication post-CABG.

## Supplemental Material

sj-docx-1-aan-10.1177_02184923251321541 - Supplemental material for Traumatic chylothorax management post-coronary artery bypass grafting – A systematic review Supplemental material, sj-docx-1-aan-10.1177_02184923251321541 for Traumatic chylothorax management post-coronary artery bypass grafting – A systematic review by Gavin John Carmichael, Duron Prinsloo, Connor Bentley, Rodan Prinsloo, Joshua G Kovoor, Mathew O Jacob and Aashray Gupta in Asian Cardiovascular and Thoracic Annals

## References

[1] McGrath EE Blades Z Anderson PB . Chylothorax: aetiology, diagnosis and therapeutic options. Respir Med 2010; 104: 1–8. 19766473 10.1016/j.rmed.2009.08.010

[2] Nair SK Petko M Hayward MP . Aetiology and management of chylothorax in adults. Eur J Cardiothorac Surg 2007; 32: 362–369. 17580118 10.1016/j.ejcts.2007.04.024

[3] Xiao Y Chen Y Huang R , et al. Incidence, risk factors, and outcomes of chylothorax after cardiac procedure in the United States. Heliyon 2024; 10: e29054. 10.1016/j.heliyon.2024.e29054 PMC11024541 38638975

[4] Bryant AS Minnich DJ Wei B , et al. The incidence and management of postoperative chylothorax after pulmonary resection and thoracic mediastinal lymph node dissection. Ann Thorac Surg 2014; 98: 232–235. discussion 5–7. 24811982 10.1016/j.athoracsur.2014.03.003

[5] Merrigan BA Winter DC O’Sullivan GC . Chylothorax. Br J Surg 1997; 84: 15–20. 9043440

[6] Loop FD Lytle BW Cosgrove DM , et al. Influence of the internal-mammary-artery graft on 10-year survival and other cardiac events. N Engl J Med 1986; 314: 1–6. 3484393 10.1056/NEJM198601023140101

[7] Aldea GS Bakaeen FG Pal J , et al. The society of thoracic surgeons clinical practice guidelines on arterial conduits for coronary artery bypass grafting. Ann Thorac Surg 2016; 101: 801–809. 26680310 10.1016/j.athoracsur.2015.09.100

[8] Power R Smyth P Donlon NE , et al. Management of chyle leaks following esophageal resection: a systematic review. Dis Esophagus 2021; 34. 10.1093/dote/doab012 PMC8597908 33723611

[9] Page MJ McKenzie JE Bossuyt PM , et al. The PRISMA 2020 statement: an updated guideline for reporting systematic reviews. Br Med J 2021: n71. 10.1136/bmj.n71 PMC8005924 33782057

[10] Moola S Munn Z Tufanaru C , et al. Chapter 7: Systematic reviews of etiology and risk. In: Joanna Briggs institute reviewer's manual, 2017, pp. 3–6.

[11] Innovation VH . Covidence. www.covidence.org.2024.

[12] Altun G Pulathan Z Kutanis D , et al. Conservative management of chylothorax after coronary artery bypass grafting. Tex Heart Inst J 2015; 42: 148–151. 25873827 10.14503/THIJ-13-3532 PMC4382882

[13] Deguchi K Yamauchi T Maeda S , et al. Chylothorax after coronary artery bypass grafting using the right internal thoracic artery. Gen Thorac Cardiovasc Surg 2015; 63: 416–421. 23921965 10.1007/s11748-013-0303-8

[14] El-Farra MH Pham N Smith J , et al. Treatment of Chylothorax after coronary artery bypass grafting. Ann Thorac Surg 2021; 112: e349–ee52. 10.1016/j.athoracsur.2021.02.057 33689740

[15] Hoskote SS Devarapally SR Dasgupta R , et al. Fatal pulmonary embolism complicating a postoperative chylothorax despite adequate thromboprophylaxis. Blood Coagul Fibrinolysis 2013; 24: 887–889. 23751608 10.1097/MBC.0b013e3283626266

[16] Kanna S Arora S Goel H , et al. Chylothorax after coronary artery bypass grafting: is it always early? J Card Surg 2021; 36: 3402–3404. 34091950 10.1111/jocs.15686

[17] Khan I Khan ZM Refy AE , et al. Chylothorax after coronary artery bypass surgery. Report of a case and review of the literature. J Saudi Heart Assoc 2020; 32: 194–199. 33154915 10.37616/2212-5043.67 PMC7640546

[18] Mukherjee K Chakrabarty U Dasbakshi K , et al. Management of Chylothorax after coronary artery bypass grafting: two case reports and review of literature. Indian J Chest Dis Allied Sci 2016; 58: 131–134. 30182684

[19] Sabzi F Yaghoubi A . The combination of breast necrosis and chylothorax following the OPCAB. J Cardiovasc Thorac Res 2016; 8: 88–90. 27493707 10.15171/jcvtr.2016.18 PMC4972404

[20] Vazhev Z Dimitrov K Stoev H . Chylothorax after cardiac surgery. Acta Med Bulg 2020; 47: 38–42.

[21] Waikar HD Kamalaneson P Mohamad Zamri MS , et al. Chylothorax after off-pump coronary artery bypass graft surgery: management strategy. Ann Card Anaesth 2018; 21: 300–303. 30052221 10.4103/aca.ACA_212_17 PMC6078034

[22] Waliany S Chandler J Hovsepian D , et al. Yellow nail syndrome with chylothorax after coronary artery bypass grafting. J Cardiothorac Surg 2018; 13: 93. 30201014 10.1186/s13019-018-0784-8 PMC6131875

[23] Marts BC Naunheim KS Fiore AC , et al. Conservative versus surgical management of chylothorax. Am J Surg 1992; 164: 532–534; discussion 4–5. 1443383 10.1016/s0002-9610(05)81195-x

[24] Binkert CA Yucel EK Davison BD , et al. Percutaneous treatment of high-output chylothorax with embolization or needle disruption technique. J Vasc Interv Radiol 2005; 16: 1257–1262. 16151069 10.1097/01.rvi.0000167869.36093.43

[25] Reisenauer JS Puig CA Reisenauer CJ , et al. Treatment of postsurgical chylothorax. Ann Thorac Surg 2018; 105: 254–262. 29132697 10.1016/j.athoracsur.2017.07.021

[26] Pego-Fernandes PM Ebaid GX Nouer GH , et al. Chylothorax after myocardial revascularization with the left internal thoracic artery. Arq Bras Cardiol 1999; 73: 383–390. 10754592

[27] Ofori-Asenso R Liew D Martensson J , et al. The frequency of, and factors associated with prolonged hospitalization: A multicentre study in Victoria, Australia. J Clin Med 2020; 9. 10.3390/jcm9093055 PMC7564707 32971851

[28] Helin RD Angeles ST Bhat R . Octreotide therapy for chylothorax in infants and children: a brief review. Pediatr Crit Care Med 2006; 7: 576–579. 16878051 10.1097/01.PCC.0000235256.00265.C8

[29] Rosti L De Battisti F Butera G , et al. Octreotide in the management of postoperative chylothorax. Pediatr Cardiol 2005; 26: 440–443. 16374694 10.1007/s00246-004-0820-4

[30] Schild HH Strassburg CP Welz A , et al. Treatment options in patients with chylothorax. Dtsch Arztebl Int 2013; 110: 819–826. 24333368 10.3238/arztebl.2013.0819 PMC3865492

[31] Shostak E Tibbetts AV Bassett JM , et al. Management of recurrent pleural effusions in patients who fail thoracoscopic talc pleurodesis. Chest 2010; 138: 344A.

[32] Parker J Mesia C Moulick A , et al. Efficacy of chemical Pleurodesis with doxycycline for Chylous pleural effusion after cardiac surgery. Crit Care Med 2013; 42.

[33] Maldonado F Cartin-Ceba R Hawkins FJ , et al. Medical and surgical management of chylothorax and associated outcomes. Am J Med Sci 2010; 339: 314–318. 20124878 10.1097/MAJ.0b013e3181cdcd6c

[34] Gómez FM Baetens TR Santos E , et al. Interventional solutions for post-surgical problems: a lymphatic leaks review. CVIR Endovasc 2024; 7: 61. 39126551 10.1186/s42155-024-00473-3 PMC11316727

[35] Cope C Kaiser LR . Management of unremitting chylothorax by percutaneous embolization and blockage of retroperitoneal lymphatic vessels in 42 patients. J Vasc Interv Radiol 2002; 13: 1139–1148. 12427814 10.1016/s1051-0443(07)61956-3

[36] Itkin M Krishnamurthy G Naim MY , et al. Percutaneous thoracic duct embolization as a treatment for intrathoracic chyle leaks in infants. Pediatrics 2011; 128: e237–e241. 10.1542/peds.2010-2016 21646254

[37] Kim PH Tsauo J Shin JH . Lymphatic interventions for chylothorax: a systematic review and meta-analysis. J Vasc Interv Radiol 2018; 29: 194–202. 29287962 10.1016/j.jvir.2017.10.006

[38] Bhatnagar M Fisher A Ramsaroop S , et al. Chylothorax: pathophysiology, diagnosis, and management – a comprehensive review. J Thorac Dis 2024; 16: 1645–1661. 38505027 10.21037/jtd-23-1636 PMC10944732

[39] Tasoglu I Lafci G Sahin S , et al. Chylomediastinum following mitral valve replacement. Thorac Cardiovasc Surg 2012; 60: 480–481. 21692018 10.1055/s-0030-1271149

[40] Shah RD Luketich JD Schuchert MJ , et al. Postesophagectomy chylothorax: incidence, risk factors, and outcomes. Ann Thorac Surg 2012; 93: 897–903. 22245587 10.1016/j.athoracsur.2011.10.060 PMC3430511

